# Supplementary material for: Identification of a pocket factor that is critical to Zika virus assembly
Source: Nat Commun. 2020 Oct 2;11:4953. doi: 10.1038/s41467-020-18747-4 (PMC7532219; doi:10.1038/s41467-020-18747-4)
Supplement: Supplementary file 1 — Supplementary Information [file 41467_2020_18747_MOESM1_ESM.pdf]

Supplementary information

Incorporation of a novel pocket factor is critical to Zika virus assembly

DiNunno et al

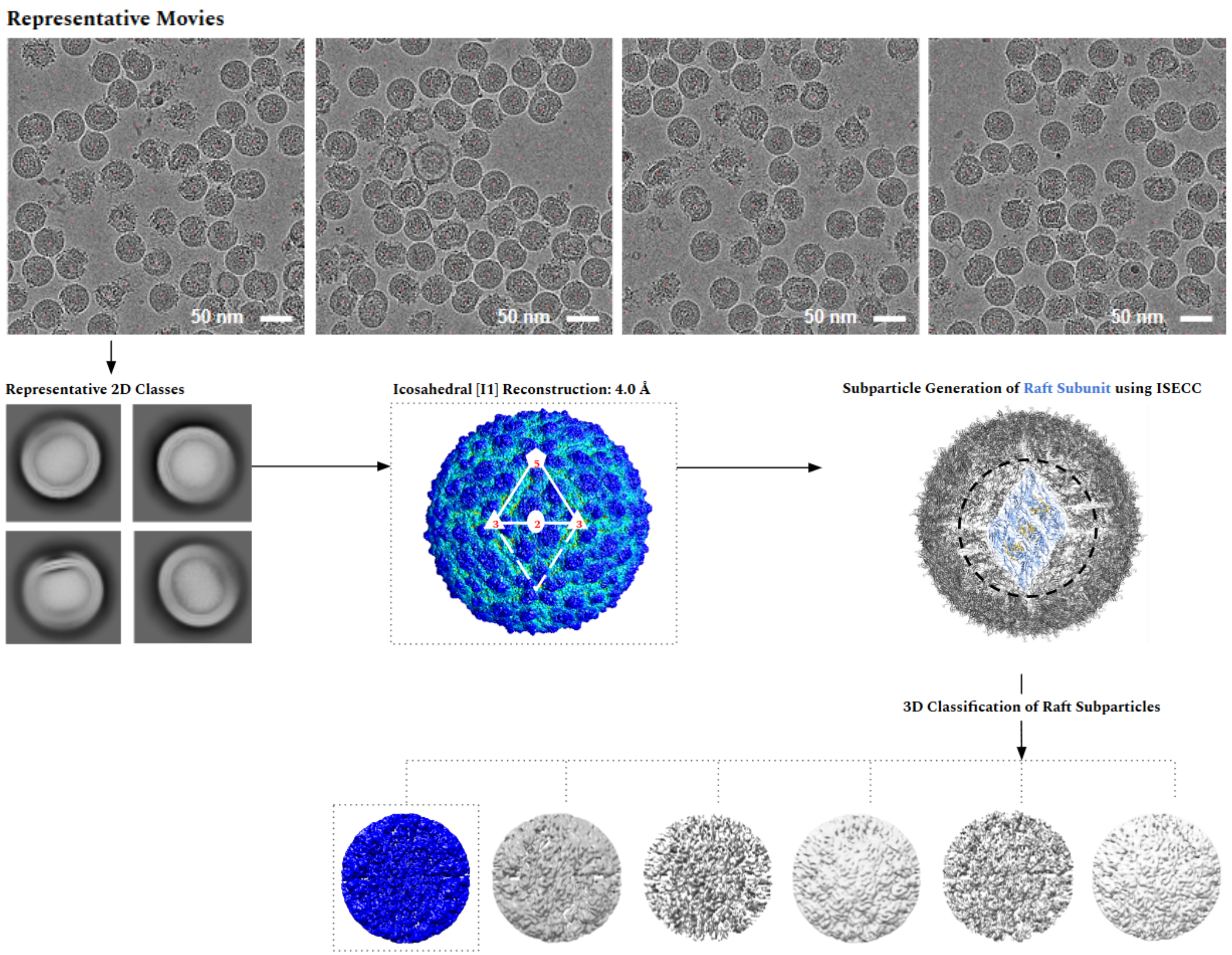

Supplementary Figure 1. Local reconstruction workflow of MR-766 ZIKV using ISECC to isolate raft subunits. The 4.0 Å icosahedrally averaged map was used to designate the subvolume containing three E-M dimer pairs. These subvolumes were used for 3D classification that resulted in six classes that varied largely by resolution. The highest resolution class (blue) proceeded to refinement and achieve the final resolution of 3.4 Å.

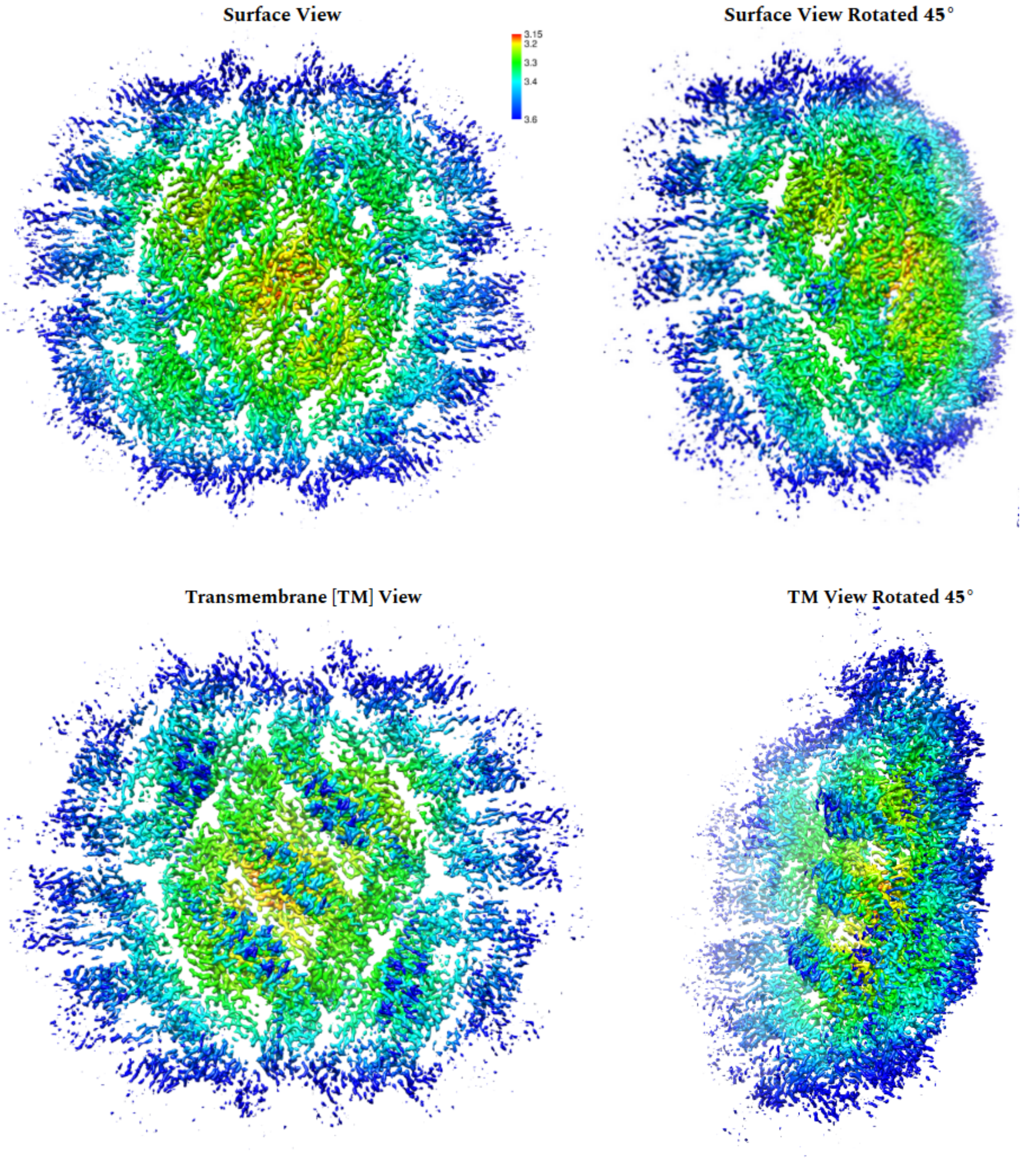

Supplementary Figure 2. Local resolution mapping of the subvolume. Local resolution was performed using RELION and is presented with four different views, A) top down, B) rotated 45° to the right, C) Transmembrane (TM) view, and D) TM view rotated 45° to the right. The surface exposed regions of E protein reached the highest local resolution of about 3.2 Å, as demonstrated by the local resolution implementation in RELION.

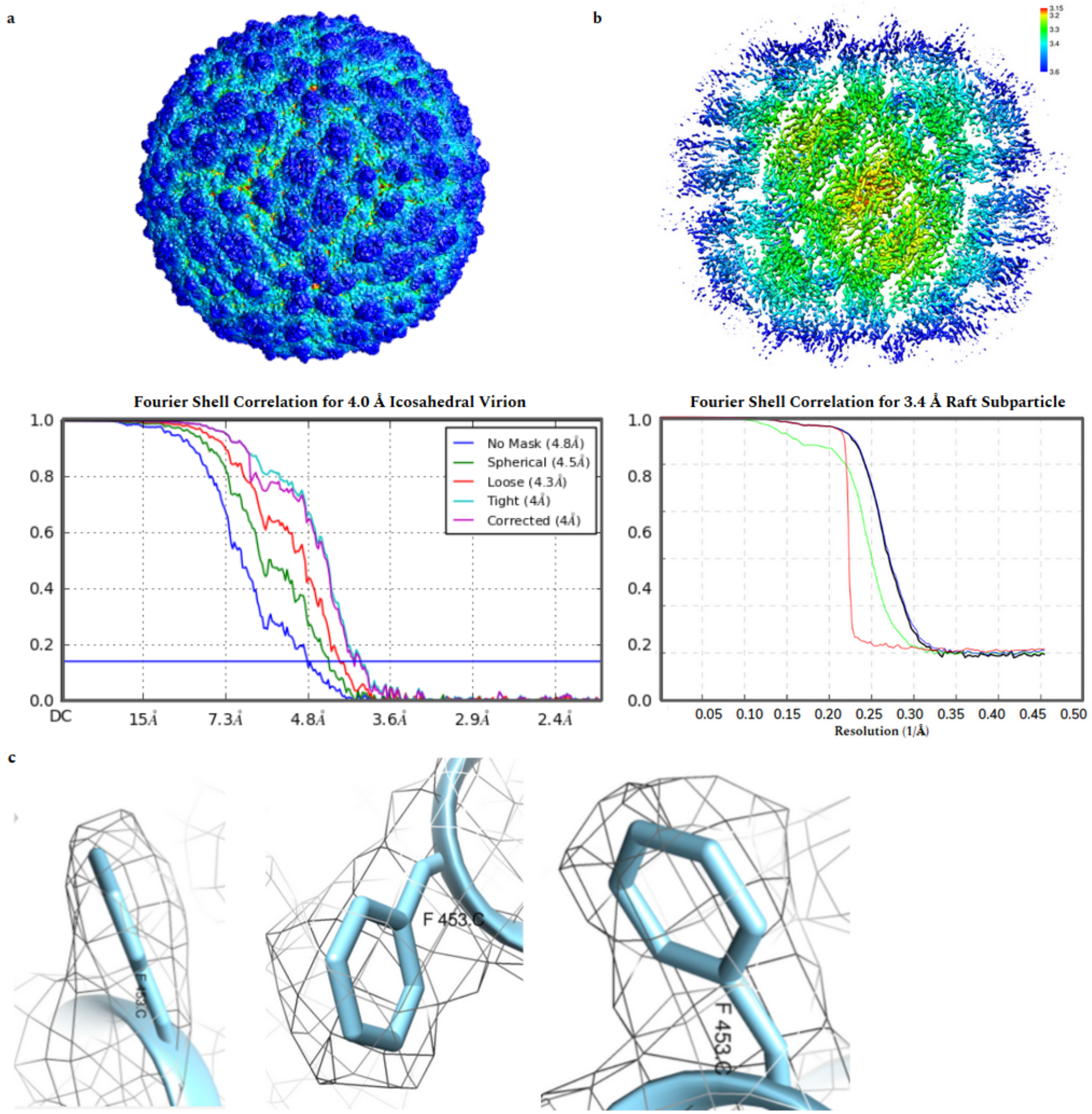

Supplementary Figure 3. Subvolume reconstruction of ZIKV improves local resolution to 3.4 Å. (A) The icosahedrally averaged ZIKV achieved a resolution of 3.99Å according to FSC resolution determination. (B) Application of localized reconstruction methods improved the map by allowing the selection of the mature regions of the virus from the heterogeneous particles (top), which achieved a final resolution of 3.4 Å with the beta-sheet regions of E glycoprotein reaching resolutions of 3.1 Å. (C) A magnified view of the electron density map fitted with the ribbon diagram of the E protein displays the impact of local reconstruction to a local resolution of 3.4 Å (bottom) in mesh diagram with F453.

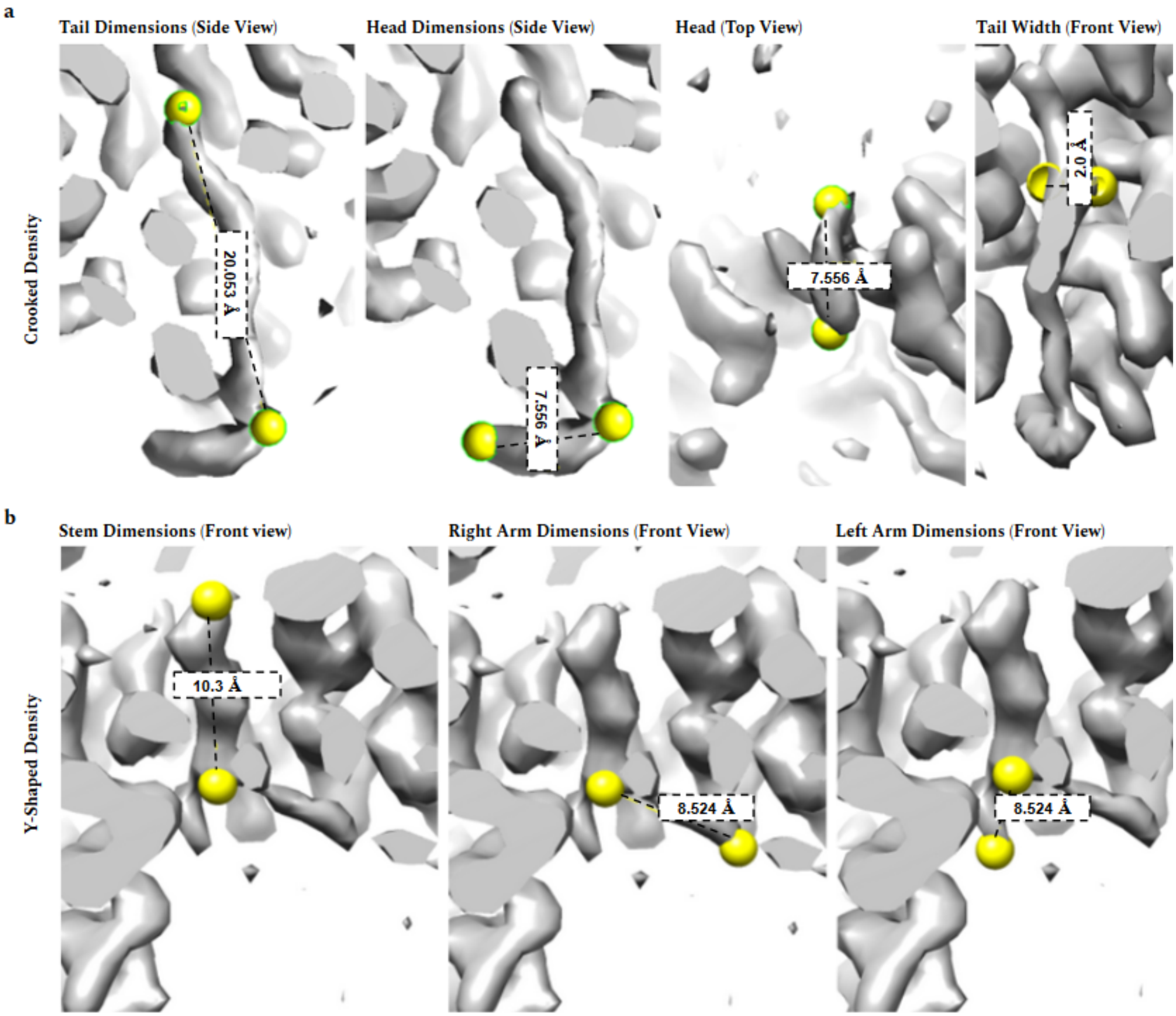

Supplementary Figure 4. Dimensions of the unfilled densities. (A) The dimensions of the crooked lipid in the cryo EM density map were measured using Chimera and found to correspond to a kinked lipid of approximately 28 Å long, 20 Å across the tail, 7.5 Å across the head, and 2 Å in width. (B) The Y-shaped lipid was found to measure 10 Å at the stem and 8 Å by 5 Å in the arms.

M Protein: Alignment of Amino Acids 20-76

T57 S58

```
TBEV       WLEGDSLRTHLTRVEGWVWKNKILTLAVIAVVWLTVESVVTRIAVVVVLLCLAPVYAS
YFV        WMTGRMGERQLQKIERWFVRNPFFAVTALTIAYLVGSNMTQRVVIALLVLAVGPAYSA
Denv-4     WMSSEGAWKHAQRVESWILRNPGFALLAGFMAYMIGQTGIQRTVFFVLMMLVAPSYGM
DENV-2     WMSSEGAWKHVQRIETWILRHPGFTMMAAILAYTIGTTHFQRALIFILLTAVTPSMTM
DENV-1     WMSSEGAWKQIQRVETWALRHPGFTVIALFLAHAIGTSITQKGIIFILLMLVTPSMAM
Denv-3     WMSAEGAWRQVEKVETWALRHPGFTILALFLAHYIGTSLTQKVVIFILLMLVTPSMTM
ZIKV       WLESREYTKHLIKVENWIFRNPGFALVAVAIAWLLGSSTSQKVIYLVMILLIAPAYSI
SLEV       WLDTVKTTKYLTKVENWVLRNPGYALVALAIGWMLGSNNTQRVVFVIMLMLIAPAYSF
WNV        WMDSTKATRYLVKTESWILRNPGYALVAAVIGWMLGSNTMQRVVFVVLLLLVAPAYSF
JEV        WLDSTKATRYLMKTENWIIRNPGYAFLAATLGWMLGSNNGQRVVFTILLLLVAPAYSF
MVEV       WLDSTKATRYLTKTENWIIRNPGYALVAVVLGWMLGSNTGQKVIFTVLLLLVAPAYSF
           *.           . * *  ::   :. .  :        .   :    :::  : *
```

E Protein: Alignment of Amino Acids 429-504

H446 F449 F453 W474 F497

```
TBEV       WDFGSTGGFLTSVGKALHTVLGGAFNSLFGGVGFLPKILMGVVLAWLGLNMRNPTMSMGFLLAGGLVLAMTLGVGA
YFV        WDFSSAGGFFTSVGKGIHTVFGSAFQGLFGGLNWITKVIMGAVLIWVGINTRNMTMSMSMILVGVIMMFLSLGVGA
Denv-4     WDFGSVGGLFTSLGKAVHQVFGSVYTTMFGGVSWMIRILIGFLVLWIGTNSRNTSMAMTCIAVGGITLFLGFTVQA
DENV-2     WDFGSLGGVFTSIGKALHQVFGAIYGAAFSGVSWTMKILIGVIITWIGMNSRSTSLSVTLVLVGIVTLYLGVMVQA
DENV-1     WDFGSIGGVFTSVGKLVHQIFGTAYGVLFSGVSWTMKIGIGVLLTWLGLNSRSTSLSMTCIAVGLVTLYLGVMVQA
Denv-3     WDFGSVGGVLNSLGKMVHQIFGSAYTALFSGVSWVMKIGIGVLLTWIGLNSKNTSMSFSCIAIGIITLYLGAVVQA
ZIKV       WDFGSVGGVFNSLGKGIHQIFGAAFKSLFGGMSWFSQILIGTLLVWLGLNTKNGSISLTCLALGGVMIFLSTAVSA
SLEV       WDFGSIGGVFNSIGKAVHQVFGGAFRTLFGGMSWITQGLLGALLLWMGLQARDRSISLTLLAVGGILIFLATSVQA
WNV        WDFGSVGGVFTSVGKAVHQVFGGAFRSLFGGMSWITQGLLGALLLWMGINARDRSIALTFLAVGGVLLFLSVNVHA
JEV        WDFGSIGGVFNSIGKAVHQVFGGAFRTLFGGMSWITQGLMGALLLWMGVNARDRSIALAFLATGGVLVFLATNVHA
MVEV       WDFGSVGGVFNSIGKAVHQVFGGAFRTLFGGMSWISPGLLGALLLWMGVNARDKSIALAFLATGGVLLFLATNVHA
           ***.* ** :.*:** :* ::*  :   *.*:.:      :* :: *:* : :. :::.  :  * : : :   * *
```

Supplementary Figure 5. Comparison of C-terminal sequences of M and E proteins of Flaviviruses. A multiple sequence alignment was performed with Clustal Omega to determine the conservation of residues comprising the transmembrane helices forming the lipid pocket. The residues contacting the lipid densities are shown in red.

	ZIKV	ZIKV Two-fold Sub-particles
**Data Collection and Processing**		
Magnification	X59000	X59000
Voltage (kV)	300kV	300kV
Electron Exposure (e-/Å^2)	41.7	41.7
Defocus Range (μm)	-1 to -3	-1 to -3
Pixel Size (Å)	1.11	1.11
Symmetry	Icos	C2 to C1
Initial Particle Images (no.)	124,444	
Final Particle Images (no.)	33,653	9181
Map Resolution	4.0	3.4
FSC Threshold	0.143	0.143
Refinement Validation		
RMS Deviations		
Bond Lengths (Å)		0.008
Angles (˚)		0.737
Validation		2.67
Molprobity Score		2.79
ClashScore		13.01
Ramachandran Plot		
Favored (%)		89.16
Allowed (%)		10.31
Outliers (%)		0.52

Supplementary Table 1. Cryo-EM Data Collection, refinement, and statistics.

Mutation	Forward (5'-3')	Reverse (5'-3')
M T57A	AAGCTCGGCTAGCCAAAAAGTCATATACTTGGTCATG	TTTTGGCTAGCCGAGCTTCCCAAAAGCCAGGCAATG
M S58A	AGCTCGACGGCTCAAAAAGTCATATACTTGGTCATG	TTTTTGAGCCGTCGAGCTTCCCAAAAGCCAGGCAATG
E H446A	AGGGCATTGCTCAGATTTTTGGAGCAGCCTTCAAATC	AAATCTGAGCAATGCCCTTACCCAGTGAGTTGAAC
E F449A	TCACCAGATTGCCGGAGCAGCCTTCAAATCACTGTTTG	TGCTCCGGCAATCTGGTGAATGCCCTTACCCAGTG
E F453A	AGCAGCCGCTAAATCACTGTTTGGAGGAATGTCCTG	AGTGATTTAGCGGCTGCTCCAAAAATCTGGTGAATG
E W474A	TGCTAGTGGCTTTAGGTTTGAACACAAAGAATGG	AACCTAAAGCCACTAGCAGCGTGCCTATGAGGATC
E W474F	TGCTAGTGTTTTTAGGTTTGAACACAAAGAATGG	AACCTAAAAACACTAGCAGCGTGCCTATGAGGATC
E W474H	TGCTAGTGCACTTAGGTTTGAACACAAAGAATGG	AACCTAAGTGCACTAGCAGCGTGCCTATGAGGATC
E W474Y	TGCTAGTGTATTTAGGTTTGAACACAAAGAATGG	AACCTAAATACACTAGCAGCGTGCCTATGAGGATC
E W474R	TGCTAGTGCGCTTAGGTTTGAACACAAAGAATGG	AACCTAAGCGCACTAGCAGCGTGCCTATGAGGATC
E F497A	TGATGATCGCTCTCTCCACGGCTGTTTCTGCTGATG	TGGAGAGAGCGATCATCACTCCCCCCAGGGCCAAG

Supplementary Table 2. Primers used for structure-based mutagenesis of lipid-contacting regions.
